# Lower Respiratory Tract Infections and Orofacial Clefts: A Prospective Cohort Study From the Japan Environment and Children’s Study

**DOI:** 10.2188/jea.JE20200438

**Published:** 2022-06-05

**Authors:** Yukihiro Sato, Eiji Yoshioka, Yasuaki Saijo, Toshinobu Miyamoto, Hiroshi Azuma, Yusuke Tanahashi, Yoshiya Ito, Sumitaka Kobayashi, Machiko Minatoya, Yu Ait Bamai, Keiko Yamazaki, Sachiko Itoh, Chihiro Miyashita, Atsuko Ikeda-Araki, Reiko Kishi, The Japan Environment and Children’s Study (JECS) Group

**Affiliations:** 1 Division of Public Health and Epidemiology, Department of Social Medicine, Asahikawa Medical University, Hokkaido, Japan; 2 Department of Obstetrics and Gynecology, Asahikawa Medical University, Hokkaido, Japan; 3 Department of Pediatrics, Asahikawa Medical University, Hokkaido, Japan; 4 Faculty of Nursing, Japanese Red Cross Hokkaido College of Nursing, Hokkaido, Japan; 5 Center for Environmental and Health Sciences, Hokkaido University, Hokkaido, Japan

**Keywords:** cohort study, orofacial clefts, respiratory tract infection

## Abstract

**Background:**

Lower respiratory tract infections (LRTIs) are a cause of inpatient and outpatient care among children. Although orofacial clefts seem to be associated with LRTIs, epidemiological studies are scarce on this topic. This study aimed to examine whether infants with orofacial clefts were associated with LRTIs.

**Methods:**

This prospective cohort study used data from the Japan Environment and Children’s Study, for which baseline recruitment was conducted during 2011–2014. This study included 81,535 participants. The number of infants with cleft lip and palate (CLP), cleft lip (CL), and cleft palate only (CP) was 67, 49, and 36, respectively. We defined history of LRTIs until 12 months’ age reported by their mothers as the dependent variable. Accumulated breastfeeding duration was used as a potential mediator.

**Results:**

The incidence proportion of LRTIs among the control group was 6.0%. The incidence proportion among infants with CLP, CL, and CP were 11.9%, 14.3%, and 5.6%, respectively. After adjusting for covariates, compared with the control group, infants with CLP and CL were associated with risk of LRTIs (incidence risk ratio [IRR] of CLP, 2.38; 95% confidence interval [CI], 1.30–4.36 and IRR of CL, 2.73; 95% CI, 1.40–5.33), but not ones with CP (IRR 1.08; 95% CI, 0.28–4.15). Accumulated breastfeeding duration decreased the IRR of CLP only (IRR of CLP, 2.16; 95% CI, 1.19–3.93).

**Conclusion:**

Infants with orofacial clefts aged 1 year have a potentially high incidence proportion of LRTIs. Accumulated breastfeeding duration might mediate the associations of CLP.

## INTRODUCTION

Orofacial clefts are a common congenital anomaly, with approximately 1 case per 700 live birth.^1^^,^^2^ Particularly, this congenital anomaly has a higher incidence rate (0.14%) in Japan.^1^^,^^2^ Orofacial clefts include cleft lip and cleft palate, and these conditions result from the lack of formations during embryogenesis development.^3^ As cleft lip and cleft palate can occur simultaneously or singly, orofacial clefts can be classified into three categories: cleft lip (CL), cleft palate only (CP), and cleft lip and palate (CLP).

Lower respiratory tract infections (LRTIs), mainly including pneumonia and bronchiolitis, have been a pervasive public health problem.^4^ There continues to be a considerable number of children as inpatients and outpatients due to LRTIs (the estimated number of inpatients and outpatients aged less than 1 year per day being 0.9 per 1,000 and 6.1 per 1,000, respectively, in Japan).^5^ The occurrence of LRTIs in children can still lead to a large burden on healthy life, healthcare utilization, and costs.^6^^,^^7^ Previous studies have reported infection complications of the cleft palate due to the unfused secondary palate. The cleft palate causes nasopharyngeal closure dysfunction along with nasal regurgitation and a decrease in the oral suction.^8^ Inadequate airway protection during swallowing further triggers aspiration pneumonia.^9^^,^^10^ Therefore, mothers of infants with a cleft palate have reported feeding difficulties.^10^ Although breastfeeding is a well-known preventive factor for respiratory infections, infants with a cleft palate have difficulty in breastfeeding from their mothers due to the unfused palate.^11^^,^^12^ The cleft palate may shorten the duration of breastfeeding and increase the risk of respiratory infections accordingly. Furthermore, infants with orofacial clefts tend to be born underweight.^13^^,^^14^ Due to impaired immune function from growth restriction, these infants can be at risk of hospitalization for pneumonia.^15^ Additionally, in Japan, cleft lip repair surgery is performed later than 3 months of age, and primary palatal surgery is performed between 12 and 24 months of age.^16^^,^^17^ Previous studies have reported that LRTIs can occur after cleft lip and palate surgery.^18^^,^^19^ Therefore, infants with cleft palate and cleft lip can have a high risk of LRTIs.

Although infants with oral clefts have been recognized as having risk factors for LRTIs, there seems to be a paucity of epidemiological evidence on the association between infants with orofacial clefts and LRTIs. To the best of our knowledge, only one study has reported these associations. A study of the Western Australian Register of Developmental Anomalies reported that infants with CLP and CP aged 0–2 years had an increased risk of hospitalization for any acute LRTIs (incidence rate ratio [IRR] of CLP, 2.5; 95% confidence interval [CI], 1.2–4.8, and IRR of CP, 2.2; 95% CI, 1.3–3.6). CL infants did not have this risk of hospitalization (IRR of CL, 0.5; 95% CI, 0.1–1.6).^20^ However, that study used hospitalization as the outcome, not the incidence of LRTIs; therefore, it could only include severe cases. In addition, they did not examine potential pathways. Potential pathway analyses can help confirm and refute hypotheses.^21^ Thus, this study aimed to examine whether orofacial clefts in infants ≤12 months of age were associated with the incidence of LRTIs using data from the Japan Environment and Children’s Study (JECS). We also examined whether history of surgery under general anesthesia, accumulated breastfeeding duration, and birthweight mediated the associations.

## METHODS

### Ethical approval

Written informed consent was obtained from all participants and their proxies. The JECS protocol was approved by the Institutional Review Board of epidemiological studies of the Ministry of the Environment and Ethics Committees of all participating institutions. The JECS was conducted in accordance with the Helsinki Declaration and with other nationally valid regulations.^22^

### Data sources and participants

This prospective cohort study was conducted using a dataset, named jecs-an-20180131, from the JECS, the details of which are reported elsewhere.^22^^,^^23^ The JECS is a nationwide birth cohort study that aims to evaluate various environmental effects on children’s health and development in Japan. The baseline recruitment was performed targeting early pregnant women in 15 areas between January 2011 and March 2014. Fifteen study areas were selected to cover the Japanese geographical areas from the north to the south. If individuals resided outside the study areas, then they could not participate in this survey.

**Figure 1.**
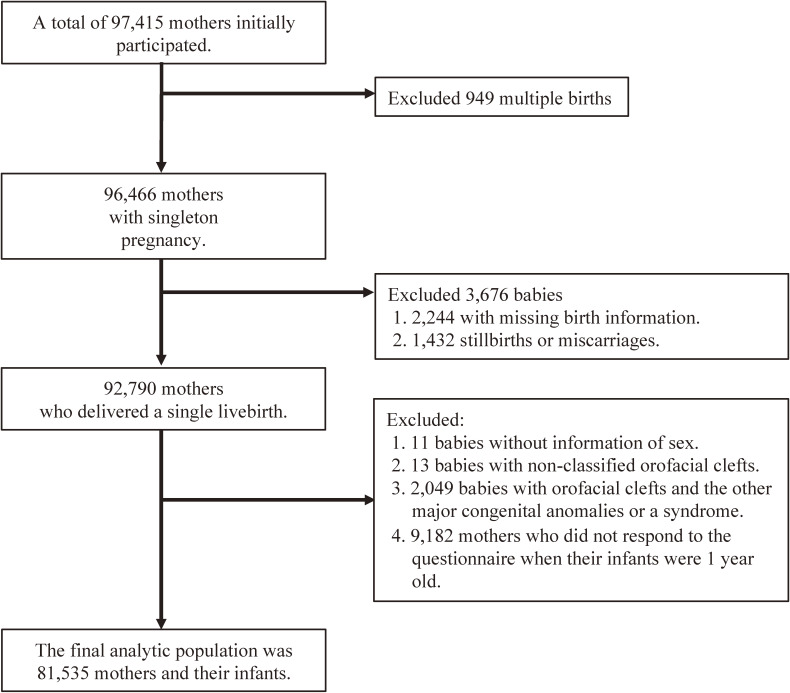
Flowchart of the selection of participants

Figure 1 shows a flowchart of the selection process of participants in this study. Because we excluded pregnant women who were participating for second time or more, 97,415 women participated in the baseline recruitment for the first time. After excluding 949 mothers who had multiple births, 96,466 mothers who had delivered a single birth remained. Among them, 2,244 had missing birth information and 1,432 had stillbirths or miscarriages; consequently, we observed a total of 92,790 single live births. We excluded 11 pairs because the sex of infants was not recorded, and 13 infants had non-classified orofacial cleft cases. In addition, to restrict nonsyndromic cases and non-affected controls, we excluded 2,049 infants with other major congenital anomalies or syndromes. We also excluded 9,182 mothers who did not respond to the questionnaire when their infants were a year old. Thus, the final analyzed population included 81,535 pairs (mother-infant).

### Independent variables: cleft lip and palate, cleft lip, and cleft palate only

We defined CLP, CL, and CP as independent variables. We obtained information on congenital anomalies from the medical record transcripts both at delivery and at 1 month of age.^24^ A previous study of JECS has described the details of the validation of the diagnosis process on congenital anomalies.^24^ We defined cases that were identified at either of the two periods after the definition of congenital anomalies in JECS.^24^ Orofacial cleft cases with an inconsistent diagnosis were defined as nonclassified orofacial clefts.^24^ Because we excluded infants with other major congenital anomalies or any syndromes, all included cases were defined as nonsyndromic. In addition, we also excluded nonclassified orofacial clefts. The control group included infants without any major congenital anomalies or syndromes.

### Dependent variable: lower respiratory tract infections until 12 months of age

We defined history of LRTIs until 12 months of age as the dependent variable. At 12 months of age, we obtained information on the history of LRTIs using a questionnaire answered exclusively by the mothers of these infants. The history of LRTIs was assessed based on the following question: “Has your child ever been diagnosed by a doctor for the following diseases, such as infectious diseases?” There was a checkbox in an infection disease section as “lower respiratory tract inflammations (such as bronchitis and pneumonia).” We defined checking the box of lower respiratory tract inflammation as having history of LRTIs until 12 months of age.

### Potential mediator variables

According to previous studies, we considered history of surgery under general anesthesia,^18^^,^^19^ birthweight,^13^^–^^15^ and the accumulated breastfeeding duration^10^^–^^12^ as potential mediator variables. Due to lack of information on objective assessments of swallowing function and aspiration, we could not include them as variables. Information on surgery was obtained using the following question at 12 months of age: “How many of the following have happened since the birth of your child?” There was a section of “Surgery with general anesthesia.” We obtained information on the number surgeries performed on the infant under general anesthesia until 12 months of age and defined the category as “none” and “at least once” accordingly. We also obtained information on birthweight from medical record transcripts at birth. Birthweight was classified as low birthweight (<2,500 g), normal birthweight (2,500–4,000 g), and high birthweight (≥4,000 g). Information on accumulated breastfeeding duration was also obtained from the checkbox question at 12 months of age. Mothers checked each box of 1 month to 12 months of age according to when they had breastfed their infant. Then, we calculated the accumulated breastfeeding duration and divided it into three categories: 0 month, 1–6 months, and 7–12 months.

### Covariates

The covariates were maternal age at delivery (<25, 25–29, 30–34, and ≥35 years old); marital status during pregnancy (married and others); maternal active smoking status during pregnancy (never smoker, former smoker, and current smoker); frequencies of maternal passive smoking status during pregnancy (none, one to six times a week, and every day); maternal educational level (high school or lower, technical junior college or technical/vocational college, and university or higher); and annual household income (<4 million yen, 4–6 million yen, 6–8 million yen, and >8 million yen); sex of the infants (male or female), season of birth (spring [March to May]; summer [June to August], autumn [September to November], and winter [December to February]); infant passive smoking status at 1 month of age (none, someone smoked in a separate room only, and someone smoked in a room with the infant); receipt of routine vaccines until 12 months of age in the National Immunization Program including Haemophilus influenzae type b, Pneumococcal, Hepatitis B virus, Diphtheria, Pertussis, Tetanus, Polio, and BCG (Bacille Calmette-Guérin) vaccines (received all vaccines and did not receive one or more); receipt of influenza virus vaccines (none or received); number of children living together at 1 year of age (none and ≥1), and attendance in nursery school at 6 months of age (yes and no).

### Statistical analysis

We conducted a Poisson regression analysis with a robust error variance to estimate IRRs for having a history of LRTIs with 95% CIs. The IRRs can be interpreted as relative risks.^25^ The adjusted model included maternal age at delivery; marital status during pregnancy; maternal active smoking during pregnancy; frequencies of maternal passive smoking status during pregnancy; maternal educational level; annual household income during pregnancy; sex of the infant; season of birth; infant passive smoking status at 1 month of age; receipt of routine vaccines in the National Immunization Program at 1 year of age; receipt of influenza virus vaccines at 1 year of age; number of children living together at 1 year of age; and attendance in nursery school at 6 months of age. Then, we confirmed whether the directions of possible mediator variables matched to our hypothesis using stratified analyses (eTable 1). Therefore, we added only accumulated breastfeeding duration in the adjusted model to confirm the potential pathway. The percentage change by each potential mediator variable was calculated using the formula (IRR_adjusted model_ − IRR_adjusted model with a potential mediator variable_)/(IRR_adjusted model_ − 1) × 100.^26^ Two-sided *P*-values <0.05 were considered to indicate statistical significance.

Because the data set had missing information, the k-nearest neighbor imputation method (the R package “DMwR”) was used based on missing data at random for variables.^27^ eTable 2 shows the number of missing data for each variable. We also conducted an available-case analysis to confirm the validity of the results after imputation. All analyses were conducted using R (version 3.5.2; R Foundation for Statistical Computing, Vienna, Austria) on macOS.

## RESULTS

This study included 81,535 participants. The median maternal age at delivery was 31.0 (the first and third quantiles were 28.0 and 35.0), and the proportion of female infants was 48.8%. The characteristics of the participants according to the type of orofacial clefts are listed in Table 1. The number of infants with CLP, CL, and CP was 67, 49, and 36, respectively. Among the control group, the proportion of patients with history of surgery under general anesthesia was 1.1%. The proportion of infants with CLP, CL, and CP was 87.9%, 85.4%, and 6.1%, respectively. The proportion of low birthweight cases in the control group was 7.8%. The proportion of infants with low birthweight among those with CLP, CL, and CP was 17.9%, 22.4%, and 8.3%, respectively. Accumulated breastfeeding duration among 76.3% of infants in the control group was 7–12 months. Among 65.7% of infants with CLP, 69.4% of infants with CL, and 41.7% of infants with CP, accumulated breastfeeding duration was 1–6 months, 7–12 months, and 1–6 months, respectively. In the control group, the proportion of infants with an incidence of LRTIs until 12 months of age was 6.0%, and this incidence proportion among infants with CLP, CL, and CP was 11.9%, 14.3%, and 5.6%, respectively.

**Table 1.** Characteristics of the participants according to the type of orofacial clefts

		Control group		Infants with CLP		Infants with CL		Infants with CP	
		(*n* = 81,383)		(*n* = 67)		(*n* = 49)		(*n* = 36)	
		*n*	%	*n*	%	*n*	%	*n*	%
**Independent variables**									
Maternal age at delivery, years	<25	7,256	8.9	6	9.0	4	8.2	1	2.8
	25–29	22,264	27.4	24	35.8	9	18.4	15	41.7
	30–34	29,251	35.9	23	34.3	21	42.9	11	30.6
	≥35	22,608	27.8	14	20.9	15	30.6	9	25.0
Marital status during pregnancy	Married	77,156	95.8	64	95.5	46	95.8	34	94.4
	Others	3,407	4.2	3	4.5	2	4.2	2	5.6
Maternal active smoking during pregnancy	Never smoker	47,941	59.7	38	56.7	25	52.1	23	63.9
	Former smoker	29,111	36.2	28	41.8	19	39.6	12	33.3
	Current smoker	3,284	4.1	1	1.5	4	8.3	1	2.8
Frequencies of maternal passive smoking status during pregnancy	None	40,681	50.5	29	43.3	21	43.8	20	55.6
	One to six times a week	25,762	32.0	26	38.8	15	31.2	12	33.3
	Every day	14,093	17.5	12	17.9	12	25.0	4	11.1
Maternal educational attainment	High school or lower	27,928	34.7	32	48.5	15	31.2	12	33.3
	Technical junior college or technical/vocational college	34,324	42.7	16	24.2	25	52.1	18	50.0
	University or higher	18,158	22.6	18	27.3	8	16.7	6	16.7
Annual household income during pregnancy	<4 million yen	29,579	39.3	21	32.8	19	45.2	12	34.3
	4–6 million yen	25,165	33.4	22	34.4	12	28.6	11	31.4
	6–8 million yen	12,203	16.2	14	21.9	6	14.3	8	22.9
	>8 million yen	8,341	11.1	7	10.9	5	11.9	4	11.4
Sex of the infant	Male	41,669	51.2	46	68.7	29	59.2	15	41.7
	Female	39,714	48.8	21	31.3	20	40.8	21	58.3
Season of birth	Spring (March to May)	18,967	23.3	20	29.9	8	16.3	6	16.7
	Summer (June to August)	20,820	25.6	12	17.9	9	18.4	11	30.6
	Autumn (September to November)	22,490	27.6	20	29.9	22	44.9	11	30.6
	Winter (December to February)	19,106	23.5	15	22.4	10	20.4	8	22.2
Infant passive smoking status at one month of age	None	39,216	48.6	32	49.2	18	37.5	23	63.9
	Someone smoked in a separate room only	39,655	49.1	32	49.2	29	60.4	13	36.1
	Someone smoked in a room with the infant	1,863	2.3	1	1.5	1	2.1	0	0.0
Receiving routine vaccines in the National Immunization Program at 1 year of age^a^	Did not receive one or more	27,971	34.4	27	40.3	24	49.0	13	36.1
	Received all vaccines	53,412	65.6	40	59.7	25	51.0	23	63.9
Receiving influenza virus vaccines at 1 year of age	None	66,694	82.0	59	88.1	43	87.8	28	77.8
	Received	14,689	18.0	8	11.9	6	12.2	8	22.2
Number of children living together at 1 year of age	None	39,750	48.8	40	59.7	20	40.8	20	55.6
	≥1	41,633	51.2	27	40.3	29	59.2	16	44.4
Attending nursery school at 6 months of age	Yes	21,464	26.5	11	16.4	6	12.2	9	25.0
	No	59,571	73.5	56	83.6	43	87.8	27	75.0
**Potential mediators**									
History of any surgery under general anesthesia	None	67,686	98.9	8	12.1	7	14.6	31	93.9
	At least once	759	1.1	58	87.9	41	85.4	2	6.1
Birthweight	Low birthweight (<2,500 g)	6,307	7.8	12	17.9	11	22.4	3	8.3
	Normal birthweight (2,500–4,000 g)	74,332	91.4	55	82.1	37	75.5	33	91.7
	High birthweight (≥4,000 g)	696	0.9	0	0.0	1	2.0	0	0.0
Accumulated breastfeeding duration	0 month	2,124	2.6	10	14.9	3	6.1	9	25.0
	1 to 6 months	17,171	21.1	44	65.7	12	24.5	15	41.7
	7 to 12 months	62,088	76.3	13	19.4	34	69.4	12	33.3
**Dependent variable**									
Lower respiratory tract infections until 12 months of age		4,863	6.0	8	11.9	7	14.3	2	5.6

CL, cleft lip; CLP, cleft lip and palate; CP, cleft palate only.

^a^Routine vaccines in the National Immunization Program included *Haemophilus influenzae* type b, Pneumococcal, Hepatitis B virus, Diphtheria, Pertussis, Tetanus, Polio, and BCG vaccines.

Table 2 shows the associations between infants with orofacial clefts and LRTIs after imputation. In the crude model, compared with non-affected controls, infants with CLP and CL were significantly associated with LRTIs (IRR of infants with CLP, 2.00; 95% CI, 1.04–3.83 and IRR of infants with CL, 2.39; 95% CI, 1.20–4.75). Infants with CP was not significantly associated with LRTIs (IRR of infants with CP, 0.93; 95% CI, 0.24–3.58). The adjusted model showed that, compared with the control group, infants with CLP and CL were associated with LRTIs (IRR of infants with CLP, 2.38; 95% CI, 1.30–4.36 and IRR of infants with CL, 2.73; 95% CI, 1.40–5.33), but not infants with CP (IRR of infants with CP, 1.08; 95% CI, 0.28–4.15). The adjusted model with accumulated breastfeeding duration showed only a decreased IRR of infants with CLP (IRR of infants with CLP, 2.16; 95% CI, 1.19–3.93 and percentage change = 15.9%; IRR of infants with CL, 2.74; 95% CI, 1.41–5.34 and percentage change = −0.6%; IRR of infants with CP, 1.04; 95% CI, 0.27–3.98 and percentage change = 50.0%). The results from an available-case analysis were consistent with those after imputation (eTable 3).

**Table 2.** Associations between orofacial cleft status and lower respiratory tract infections after imputation

	*n*	Incidence	Incidence proportion (%)	Crude model		Adjusted Model^a^		Adjusted Model^a^ + Accumulated breastfeeding duration		Percentage change by accumulated breastfeeding duration^b^
				(*n* = 81,535)		(*n* = 81,535)		(*n* = 81,535)		
				IRR	95% CI	IRR	95% CI	IRR	95% CI	
Control group (reference)	81,383	4,863	6.0	1.00	—	1.00	—	1.00	—	
Infants with CLP	67	8	11.9	2.00	1.04, 3.83	2.38	1.30, 4.36	2.16	1.19, 3.93	15.9
Infants with CL	49	7	14.3	2.39	1.20, 4.75	2.73	1.40, 5.33	2.74	1.41, 5.34	−0.6
Infants with CP	36	2	5.6	0.93	0.24, 3.58	1.08	0.28, 4.15	1.04	0.27, 3.98	50.0

CI, confidence interval; CL, cleft lip; CLP, cleft lip and palate; CP, cleft palate only; IRR, incidence risk ratio.

^a^Adjusted Model included factors such as maternal age at delivery, marital status during pregnancy, maternal active smoking during pregnancy, frequencies of maternal passive smoking status during pregnancy, maternal educational attainment, annual household income during pregnancy, sex of the infant, season of birth, infant passive smoking status at one month of age, receiving routine vaccines in the National Immunization Program at 1 year of age, receiving influenza virus vaccines at 1 year of age, number of children living together at 1 year of age, and attending nursery school at 6 months of age.

^b^The percentage change by each potential mediator variable was calculated using the formula (IRR_adjusted model_ − IRR_adjusted model with a potential mediator variable_)/(IRR_adjusted model_ − 1) * 100.

The imputation was conducted using the k-nearest neighbor imputation method.

## DISCUSSION

Among the control group, the incidence of LRTIs was 6.0%. Among infants with CLP, CL, and CP, the incidence of LRTIs was 11.9%, 14.3%, and 5.6%, respectively. A history of surgery and birth weight were excluded due to the inconsistent directions of the associations. Accumulated breastfeeding duration decreased the IRR of CLP only. This study shows that infants with CLP and CL had an increased risk of LRTIs after adjusting for covariates, while infants with CP did not.

This study showed that only infants with CP were not associated with increased risk. However, a previous study reported that infants with CLP and CP were associated with the risk of hospitalization for any acute LRTIs, but not CL.^20^ There could be potential reasons for this contradictory result. Moreover, both the current study and the previous study did not include the severity of orofacial clefts. In Table 1, the proportion of infants with low birthweight was relatively higher among CLP and CL cases than among CP cases. Previous studies reported low birthweight among CLP and CP cases but not among CL cases.^13^^,^^14^ This result might reflect that the current CP cases included milder cases, such as Veau I, which might avoid aspiration and subsequent LRTIs. In addition, our study included only infants aged 12 months. In Japan, cleft palate surgery is generally performed after 12 months of age. As our study participants were 12 months of age, they should not be exposed to the risk of complications after surgery. Indeed, 93.9% of infants with CP were not experienced any surgery under general anesthesia. There is still a possibility that CP infants would have an increased risk after 12 months of age. In addition, the CL cases might include more severe cases such as a complete cleft lip. Previous studies showed no significant association between CL and low birthweight.^13^^,^^14^ However, in this study, there was a larger proportion of infants with low birthweight among the CL cases than among the non-affected controls (22.4% vs 7.8%). Further studies including a detailed classification of clefts are hence, needed to verify the validity of these results.

Although we conducted a mediation analysis, there was a limitation in the sample size. The models showed significant associations, yet they also presented with wide CIs, implying that the current results had a high degree of uncertainty. For example, the percentage change in infants with CP by accumulated breastfeeding duration was 50%, and the IRR changed from 1.08 to 1.04 with wide CIs. When interpreting the mediation analysis, we should note a high degree of uncertainty in the results from the mediation analyses.

We considered four pathways: infectious complications of cleft surgery, low birthweight, shorter duration of breastfeeding, and aspiration due to the cleft palate. First, we could not conduct the mediation analysis using history of surgery due to the inconsistent directions of the associations. eTable 1 shows the low incidence of LRTIs among infants with orofacial clefts who had a history of surgery. This result means that history of surgery among infants with CLP or CL seems to be associated with the decreased risk of LRTIs. This is inconsistent with the results from recent studies, which reported a 1.9–8.9% increase in respiratory complications among orofacial cleft patients after repair surgery.^18^^,^^19^ In this study, the time sequence between the outcome and surgery was lacked. Moreover, we could not collect details on surgery for clefts. Therefore, it is difficult to determine whether a repair surgery for clefts mediated the associations between infants with oral clefts and LTRIs. Further studies should the detailed information on surgery and severity for cleft lip. Second, birthweight also showed the inconsistent directions of the associations, although 17.9% of infants with CLP and 22.4% of infants with CL were born with low birthweight. eTable 1 shows that nonaffected infants with low birthweight had a low risk of LRTIs, whereas infants with orofacial clefts who were low birthweight had a high risk of LRTIs. Birthweight might not mediate the associations. Third, the IRRs of infants with CLP decreased after adjusting for accumulated breastfeeding duration, whereas those of infants with CL did not (percentage change in infants with CPL = 15.9% and that in infants with CL = −0.6%). Breastfeeding can promote the infant’s immune function and development of the respiratory tract.^12^ Previous studies have suggested that mothers of infants with a cleft palate tend to face feeding difficulties due to nasopharyngeal closure dysfunction and poor oral suction.^10^ In this study, a shorter duration of breastfeeding was observed among CLP and CP. The duration of breastfeeding may partially explain the association between infants with CLP only and LRTIs but not between infants with CL and LRTIs. Finally, we supposed that aspiration is an important mechanism of LRTIs for cleft palate. However, our assumption seems to be inconsistent because the current results show that infants with CLP had an increased risk, while infants with CP did not. In addition, infants with CL had a higher risk of LRTIs than ones with CLP (IRR of CLP, 1.96 and IRR of CL, 2.32). To determine how cleft palate is linked to LRTIs, objective assessments of swallowing function and aspiration should be included in further studies.

This study has some limitations. First, the outcomes were reported by mothers. Although information on infections reported by their mothers has been used and validated,^28^^,^^29^ subjective reporting bias might have led to an overestimation of risk. Second, there was no information on the severity of orofacial clefts. For example, it is important to determine whether clefts involve the lip, alveolus (and primary palate), and/or secondary palate and whether the cleft phenotype is complete, incomplete, partial, unilateral, bilateral, or central.^30^ Further studies should include the information on the severity of orofacial clefts. Third, although this study included a history of surgery, the time relationship between outcome and surgery was unknown. Moreover, we could not collect details on surgery for cleft lip. As this study only included infants aged 1 year, they might not have been exposed to the risk of complications after primary cleft palate surgery. Indeed, 93.9% of infants with CP did not undergo any surgery under general anesthesia. There is still a possibility that infants with CP may have an increased risk after 12 months of age. The current results might have underestimated the risk of LRTIs in infants with CP. Fourth, although the follow-up rate was relatively high, approximately 10% of the participants were lost to follow-up. Indeed, 25 infants with orofacial clefts were lost at 1 year of age. In addition, the previous JECS study indicated that worse infant health status was a risk factor for loss to follow-up during the first year postpartum.^31^ Hence, severe LRTI cases might have been lost to follow up in this study. Fifth, the sample size of infants with orofacial clefts was relatively small. In particular, the number of infants with CP was only 36. Thus, the results of infants with CP might have been estimated with insufficient power. Finally, there was no information on preventive treatments for LRTIs. For example, a systematic review indicated the effects of palivizumab in significantly reducing the frequency of hospitalizations owing to respiratory syncytial virus infections.^32^ Further studies should also include information on preventive treatments, such as the use of palivizumab.

In conclusion, infants with CLP and CL were associated with an increased risk of LRTIs, but ones with CP were not. Further studies should, however, include objective assessments of swallowing function and aspiration accordingly.

## APPENDIX A. SUPPLEMENTARY DATA

eTable 1. Associations between orofacial cleft status and lower respiratory tract infections stratified by potential mediator variables

eTable 2. Missing values for each variable

eTable 3. Associations between orofacial cleft status and lower respiratory tract infections from available-case analysis
