# Supplementary material for: Lower Respiratory Tract Infections and Orofacial Clefts: A Prospective Cohort Study From the Japan Environment and Children’s Study
Source: J Epidemiol. 2022 Jun 5;32(6):270–6. doi: 10.2188/jea.JE20200438 (PMC9086306; doi:10.2188/jea.JE20200438)
Supplement: Supplementary file 1 [file je-32-270-s001.pdf]

eTable 1. Associations between orofacial cleft status and lower respiratory tract infections stratified by potential mediator variables

			n	Incidence	Incidence proportion (%)
History of any surgery under general anesthesia	None	Control group	63,563	4,123	6.5
		Infants with CLP	6	2	33.3
		Infants with CL	6	1	16.7
		Infants with CP	29	2	6.9
	At least once	Control group	701	58	8.3
		Infants with CLP	52	6	11.5
		Infants with CL	36	5	13.9
		Infants with CP	2	0	0.0
Birthweight	Normal birthweight (2500–4000 g)	Control group	69,867	4,465	6.4
		Infants with CLP	49	6	12.2
		Infants with CL	31	6	19.4
		Infants with CP	32	1	3.1
	Low birthweight (<2500 g)	Control group	5,954	353	5.9
		Infants with CLP	10	2	20.0
		Infants with CL	10	1	10.0
		Infants with CP	2	1	50.0
	High birthweight (≥4000 g)	Control group	654	42	6.4
		Infants with CLP	-	-	-
		Infants with CL	1	0	0.0
		Infants with CP	-	-	-
Accumulated breastfeeding duration	0 month	Control group	1,999	125	6.3
		Infants with CLP	10	0	0.0
		Infants with CL	2	1	50.0
		Infants with CP	8	1	12.5
	1 to 6 months	Control group	15,865	1,306	8.2

	Infants with CLP	37	7	18.9
	Infants with CL	10	2	20.0
	Infants with CP	14	1	7.1
7 to 12 months	Control group	58,656	3,432	5.9
	Infants with CLP	12	1	8.3
	Infants with CL	30	4	13.3
	Infants with CP	12	0	0.0

CL, cleft lip; CLP, cleft lip and palate; CP, cleft palate only.

eTable 2. Missing values for each variable

Variables	Number of missing		Types of variable in the imputation
	n	%	(Categorical or continuous variables)
Orofacial clefts	0	0.0	Categorical
Maternal age at delivery	4	0.0	Categorical
Marital status during pregnancy	821	1.0	Categorical
Maternal active smoking during pregnancy	1,048	1.3	Categorical
Frequencies of maternal passive smoking during pregnancy	848	1.0	Categorical
Maternal educational attainment	975	1.2	Categorical
Annual household income during pregnancy	6,106	7.5	Categorical
Sex of the infant	0	0.0	Categorical
Season of birth	0	0.0	Categorical
Infant passive smoking status at one month of age	652	0.8	Categorical
Receiving routine vaccines in the National Immunization Program at 1 year of age	0	0.0	Categorical
Receiving influenza virus vaccines at 1 year of age	0	0.0	Categorical
Number of children living together at 1 year of age	0	0.0	Categorical
Attending nursery school at 6 months of age	348	0.4	Categorical
History of any surgery under general anesthesia	12,943	15.9	Categorical
Birthweight	48	0.1	Categorical
Accumulated breastfeeding duration	0	0.0	Categorical
Lower respiratory tract infections until 12 months of age	0	0.0	Categorical

The weighted average methods were used, and the number of neighbors was defined as 3.

eTable 3. Associations between orofacial cleft status and lower respiratory tract infections from available-case analysis

				Crude model (n=81,535)		Adjusted Model[a] (n=73,365)		Adjusted Model[a] + Accumulated breastfeeding duration (n=73,365)		Percentage change by accumulated breastfeeding duration[b]
	n	Incidence	Incidence proportion (%)	IRR	95% CI	IRR	95% CI	IRR	95% CI	
Control group (reference)	81,383	4,863	6.0	1.00	-	1.00	-	1.00	-	
Infants with CLP	67	8	11.9	2.00	1.04, 3.83	2.51	1.38, 4.58	2.29	1.26, 4.15	14.6
Infants with CL	49	7	14.3	2.39	1.20, 4.75	2.39	1.05, 5.41	2.42	1.07, 5.46	-2.2
Infants with CP	36	2	5.6	0.93	0.24, 3.58	1.12	0.29, 4.28	1.06	0.28, 4.10	50.0

CI, confidence interval; CL, cleft lip; CLP, cleft lip and palate, CP, cleft palate only; IRR, incidence risk ratio.

[a]Adjusted Model included factors such as maternal age at delivery, marital status during pregnancy, maternal active smoking during pregnancy, frequencies of maternal passive smoking status during pregnancy, maternal educational attainment, annual household income during pregnancy, sex of the infant, season of birth, infant passive smoking status at one month of age, receiving routine vaccines in the National Immunization Program at 1 year of age, receiving influenza virus vaccines at 1 year of age, number of children living together at 1 year of age, and attending nursery school at 6 months of age.

[b]The percentage change by each potential mediator variable was calculated using the formula $(IRR_{\text{adjusted model}}-IRR_{\text{adjusted model with a potential mediator variable}})/(IRR_{\text{adjusted model}}-1)*100$.
